# Identifying Indicators of Readiness and Capacity for Implementing Farm‐to‐School Interventions

**DOI:** 10.1111/josh.12747

**Published:** 2019-04-01

**Authors:** Eunlye Lee, Carol Smathers, Ana C. Zubieta, Sarah Ginnetti, Anjli Shah, Darcy A. Freedman

**Affiliations:** ^1^ Department of Pediatrics, Center for Child Health and Policy Case Western Reserve University 11100 Euclid Avenue MS 6036 Cleveland OH 44106; ^2^ College of Food, Agricultural, and Environmental Sciences Ohio State University Extension, The Ohio State University 219C Parker Food Science and Technology Building, 2015 Fyffe Court, Columbus OH 43210; ^3^ Ohio SNAP‐Ed, College of Food and Environmental Sciences & Education and Human Ecology, OSU Extension and Human Sciences 315 Campbell Hall, 1787 Neil Avenue, Columbus OH 43210; ^4^ Creating Healthy Communities, Ohio Department of Health 35 E Chestnut Street, 5th Floor, Columbus OH 43215; ^5^ Prevention Research Center for Healthy Neighborhoods Case Western Reserve University 11000 Cedar Avenue, Cleveland OH 44106; ^6^ Department of Population and Quantitative Health Sciences Case Western Reserve University 11000 Cedar Avenue, Room 443, Cleveland OH 44106

**Keywords:** farm‐to‐school, community readiness, childhood obesity, fruit and vegetable consumption, healthy nutrition, nutrition education

## Abstract

**BACKGROUND:**

Farm‐to‐school interventions are recommended strategies to improve dietary behaviors among school‐aged children. Tools are needed to assess community readiness and capacity to optimize farm‐to‐school implementation. The objective of this study was to identify and prioritize factors to inform tailored farm‐to‐school implementation by practitioners working in diverse contexts.

**METHODS:**

Practitioners and community residents (N = 194) participated in semistructured interviews (N = 18) and focus groups (N = 23). Thematic analysis was conducted to identify themes and subthemes influencing farm‐to‐school implementation. The subthemes were operationalized into measureable indicators. The themes and their associated indicators were prioritized through a consensus conference with an expert panel (N = 18).

**RESULTS:**

The qualitative data analysis and consensus conference yielded 4 themes and 17 indicators associated with community readiness and capacity to implement farm‐to‐school. The themes represent school capacity, networks and relationships, organizational and practitioner capacity, and community resources and motivations.

**CONCLUSIONS:**

Findings highlight a range of indicators of community readiness and capacity needed to support farm‐to‐school implementation. Results offer guidance for tailoring intervention delivery based on levels of community, school, practitioner, and organizational readiness and capacity.

Childhood obesity rates among US children aged 2 to 19 years have tripled during the past few decades from about 5% in the 1980s to 17% from 2011 to 2014.^1^ Substantial racial and economic disparities in childhood obesity rates remain of great public health concern. For instance, African American (19.5%) and Hispanic (21.9%) children are disproportionately affected by obesity, compared to their white counterparts (14.7%).^2^ Furthermore, childhood obesity increases risk for preventable chronic diseases such as hypertension and type 2 diabetes that manifest both in the short‐term as well as over the life course.^3^

One strategy to shift childhood obesity trends is to implement interventions designed to establish healthy dietary behaviors during childhood.^4^, ^5^, ^6^ A key behavioral target for these types of interventions is improved fruit and vegetable consumption.^7^ In 2007–2010, 60% and 93% of children aged 1 to 18 years did not consume the daily recommended number of fruits and vegetables, respectively.^8^ Prevention and intervention programs to increase fruit and vegetable consumption are recommended to reverse obesity trends among children.^9^, ^10^ Obesity prevention efforts have increasingly shifted from addressing individual‐level behaviors to implementing efforts aimed at changing broader contextual factors including access to nutritious foods within community settings (ie, schools) and their role in shaping dietary behaviors among children.^11^, ^12^, ^13^

Because dietary behaviors established in childhood may continue to influence food choices in adulthood and school‐aged children spend a significant amount of time in school, policy, systems, and environmental (PSE) interventions are promoted to address contextual factors related to fruit and vegetable consumption in schools.^13^, ^14^ In particular, “farm‐to‐school” refers to a range of activities for promoting, procuring, serving, and teaching about local foods in schools.

Previous studies and program evaluations suggest that farm‐to‐school is associated with a variety of positive outcomes including increased school meal participation,^6^ increased fruit and vegetable selection,^15^ willingness to taste and preferences for fruits and vegetables,^16^ and increased fruit and vegetable intake.^6^, ^17^, ^18^ Spurred by the positive outcomes, farm‐to‐school has grown dramatically, rising from fewer than 10 programs in 1998^6^ to programs in approximately 42% of school districts representing 42,587 schools and 23.6 million students nationwide in 2015.^19^

Although popularity of farm‐to‐school has increased in recent years, current evidence suggests that there are barriers to implementing these types of interventions. Barriers include budget constraints and distribution challenges;^20^ insufficient quantity of produce from local growers and producers;^21^ procurement regulations;^22^ lack of knowledge among school food service employees;^23^ and lack of time, curriculum materials, and training for teachers.^24^ Tools for systematically assessing community readiness and capacity to tailor farm‐to‐school implementation to the realities of local contexts can help mitigate these barriers.^25^, ^26^

The goal of this research was to develop this type of tool to facilitate wide scale implementation of farm‐to‐school. This study presents findings related to identifying, operationalizing, and prioritizing facilitators of and barriers to implementing farm‐to‐school within low‐resource rural and urban contexts. Details about the overall study methods, including use of both qualitative research and consensus conference, were previously published.^27^

## METHODS

This study is part of *Building Capacity for Obesity Prevention* (BCOP), a collaborative study between researchers at 2 universities and public health and community nutrition practitioners in the state of Ohio. Practitioners included representatives from the Ohio Supplemental Nutrition Assistance Program Education (SNAP‐Ed) and the Ohio Department of Health (ODH)'s Creating Healthy Communities (CHC) program. BCOP's primary goal is to optimize implementation of 4 nutrition‐related PSE interventions (ie, farmers' markets, healthy eating in childcare settings, healthy food retail, and farm‐to‐school) through the development of community readiness and capacity assessment tools.

### Participants

We used a purposive sampling procedure to select targeted geographic areas and recruit diverse study participants.^28^ Nine counties in Ohio were selected for recruitment because they had on‐the‐ground SNAP‐Ed and CHC staff to support farm‐to‐school implementation. In addition, these 9 counties represented diversity in terms of county health rankings, geographic location, adult obesity rates, and SNAP participation.

*Practitioners*. Within the targeted counties, public health and community nutrition practitioners (N = 20) working with SNAP‐Ed or supported by CHC were recruited by email. The email was sent by the contact from their respective business organization (ie, CHC practitioners received an email from ODH while SNAP‐Ed practitioners received it from Ohio State University Extension). Detailed information about the overall interview process was provided to prospective participants by the study coordinator through follow‐up emails. Informed consent was obtained from the practitioners prior to the interview. We conducted 18 solo or dyad in‐person interviews with 9 SNAP‐Ed and 11 CHC practitioners. Background information of interview participants was not collected to maintain confidentiality.

*Community residents*. Community members in targeted counties receiving or eligible to receive federal food assistance benefits and members of CHC coalitions were recruited through flyers posted within public spaces or at CHC coalition meetings. Interested participants called the study phone line to learn about and register for the study. All focus group participants provided written informed consent before joining the study. We conducted 23 focus groups with 174 participants (ie, 127 community members and 47 CHC coalition members). Approximately, 70% of the focus group participants were female and 65% self‐reported that they were receiving federal food assistance benefits at the time of data collection. Nearly 60% of the focus group participants self‐identified as white and 40% as African American.

*Expert panelists*. An expert panel (N = 18) was recruited for a face‐to‐face consensus conference to prioritize the themes and indicators. The panelists were purposively selected for their expertise in farm‐to‐school, experience in community nutrition and public health practice, and/or experience working with low‐income populations or in school settings. All procedures related to the consensus conference were approved by the Institutional Review Board. Due to the small sample size that was easy to identify based on demographic characteristics, background information of panelists was not collected.

### Instrumentation

The research team worked collaboratively to develop interview and focus group guides. The guides were developed based on existing models focused on factors associated with increasing implementation of nutrition‐related PSE interventions as well as through an extensive review of literature on best practices for key informant interviews.^29^, ^30^, ^31^, ^32^ In particular, they focused on participants' perceptions of their community's readiness for obesity prevention, with specific attentions to the aforementioned 4 PSEs approaches. The drafts were reviewed and discussed with key partners of the study composed of researchers, county‐ and state‐level public health and community nutrition practitioners, and Cooperative Extension professionals. The revisions were made based on feedbacks from these partners. The guides were tested by the research team before they conducted the interviews and focus groups. Both guides are available upon request from the corresponding author.

### Procedure

Semistructured and open‐ended in‐person and focus group interviews were conducted between April and June 2015. The interviews took place in convenient locations for participants such as community centers, practitioner offices, and health department offices. Interviews and focus groups were conducted in English by a lead moderator and a note taker and lasted between 1 and 2 hours. All interviews were conducted once. They were digitally recorded, transcribed verbatim by a third party transcriptionist, and all transcripts (N = 41) were checked for accuracy against original recordings.

### Data Analysis

All transcripts were coded by trained researchers using qualitative data analysis software (ie, ATLAS.ti, version 7).^33^ The coding process was an iterative process based on a grounded theory approach that included both inductive and deductive methods.^34^ Inductive coding was used first through “open codes” that were grounded in the real words of the participants through line‐by‐line reading of the transcripts. All open codes were also co‐coded with an associated PSE code to facilitate analysis of data relevant to farm‐to‐school. Deductive coding was informed by the initial conceptual framework that was expanded as new concepts emerged during open coding. These processes guided the development of a codebook with themes, subthemes, and definitions which was then used by the team to analyze all transcripts. Each open code was assigned to a subtheme and then to a higher‐level theme code to develop the coding structure.

The next step was the development of indicators for farm‐to‐school implementation within communities based on the themes and subthemes from the qualitative findings. The themes and subthemes were prioritized to include more common and salient concepts by applying an established threshold to select themes with at least 50 unique references and subthemes with at least of 1% of the total open codes. The selected subthemes were then operationalized into measurable indicators along with operational definitions of each theme.

### Consensus Conference and Pilot‐Testing

The themes and indicators derived from findings from qualitative data analysis were prioritized and validated through the consensus conference and a pilot‐testing. The expert panelists participated in 3 activities to assess the relevance and importance of the themes and indicators. First, panelists worked together in small groups to sort indicators into thematic piles. Second, the groups selected the top 3 indicators within each theme pile based on their perceived importance related to successful implementation of farm‐to‐school. Lastly, panelists participated in a voting process designed to assign weights related to the importance of each theme.

Based on the findings and feedback from the expert panel, the research team remapped themes and indicators. For example, one indicator related to sourcing and distribution systems was divided into 2 distinct indicators based on panelist recommendations. After the themes were remapped, the research team asked the expert panel to provide new weights for the revised set of themes via electronic voting. For each respective theme, the set of indicators was limited to include those that contributed to at least 80% of the total indicator weight (see Lee et al^27^ for more details). Finally, the indicators were pilot‐tested with new farm‐to‐school expert panelists (N = 5) to assess content and face validity.

## RESULTS

The qualitative analysis yielded 6 themes and 27 subthemes from a total of 931 open codes as the thresholds for themes and subthemes were applied. We initially developed 23 indicators based on the subthemes. Responses for all indicators were on a 5‐point Likert scale from *not at all* (0) to *extremely* (5) with a *do not know* (6) option. The consensus conference and pilot‐testing guided prioritization and further refinement to establish 4 themes and their respective 17 indicators for the final assessment tool. The final themes represent school capacity, networks and relationships, organizational and practitioner capacity, and community resources and motivations. The resulting product is the PSE Readiness Assessment and Decision Instrument (PSE READI). Table 1 presents the tool's themes and indicators with their respective standardized weights generated from the expert panelists and exemplary interview and focus group quotations that support each indicator. Higher weights indicate greater perceived relative importance.

**Table 1:** Final Themes, Indicators, and Exemplary Interview and Focus Group Quotations for Farm‐to‐School Interventions

Indicator by each theme	Standardized indicator weight‡	Exemplary interview and focus group quotations
School capacity (standardized theme weight = 0.36)†		
**1.1.** To what extent do school food service guidelines (eg, procurement, purchasing, food safety regulation) in your service area support farm‐to‐school PSE projects?	0.28	“One of the big things that makes it difficult for us to be able to do a farm‐to‐school program is that we have guidelines, state guidelines that require certain sized apples and certain sized pears and so we're looking for something that is a specific size, so that we're meeting our guidelines and it's hard for you know, farmers just to… it's hard for me to get to maybe a local orchard and say Hey, I need apples this size and that's all I can take.” (CHC Rural Focus Group)
**1.2.** To what extent are farm‐to‐school PSE projects in your service area integrated into school curriculum and activities (eg, greenhouse integrated with science class)?	0.25	“It's also exciting cause last year we worked with another high school, we actually partnered with them to construct a greenhouse and then provide all the supplies, and for growing for it, for the salad bar, and it's nice they collaborated that into the science classes as well.” (CHC Rural Interview) “School gardens…incorporated into science, math, and culture classes…” (SNAP‐Ed Rural Focus Group)
**1.3.** To what extent is there buy‐in from teachers, food service staff, custodial staff, and/or administrators to implement farm‐to‐school PSE projects in their schools?	0.19	“…it takes that commitment from a staff member in the school environment to make that [farm‐to‐school] happen.” (CHC RURAL Interview)
**1.4.** To what extent are there supportive programs and resources available to maintain school gardens during summer?	0.14	“so we try to get, um…either students volunteered or staff from the schools or um…whoever we are partnering with…and some community members as well, we're trying to get them to maintain it [school garden] while the school is out.” (CHC Rural Interview)
**1.5.** To what extent does school staff (eg, teacher, nurse, and cafeteria manager) in your service area have paid time available to work on farm‐to‐school PSE projects?	0.13	“Teachers don't have one extra minute to be doing things” (CHC Urban Focus Group) and “there's not enough time in a day for schools to do these extra projects.” (CHC Rural Interview) “…you know, cause I know how much time they [cafeteria workers] spend. They got one lady that does the salad bar down there and I know how much time she takes to get…everything ready so I can see where they're [school staff] coming from in that it would be difficult.” (CHC Rural Focus Group)
Networks and relationships (standardized theme weight = 0.30)†		
**2.1.** To what extent are there champions for farm‐to‐school PSE projects in your service area?	0.28	“…there's someone that is championing that effort [farm‐to‐school] because it's important, so we have someone willing to do that and that is the motivation and that's how they get other people on board.” (CHC Urban Focus Group)
**2.2.** To what extent are there sourcing and aggregation systems available in your service area to support access to locally produced food items at schools?	0.26	“Locally grown food…just finding local growers that can provide enough supply for a school when they are feeding 300 children, you know, that's difficult.” (SNAP‐Ed Urban Interview)
**2.3.** To what extent are there distribution systems available in your service area to support access to locally produced food items at schools?	0.26	“The schools are reluctant to search locally if they don't know the farmer can produce it and so you go through a big provider and they are going to compile it from several sources. But the distributors are really starting to look at that local so they may compile from several local resources and those are the kind of things that are being looked at.” (CHC Urban Focus Group)
**2.4.** To what extent are you connected to or partnered with organizations and/or key personnel (ie, statewide farm‐to‐school programs, Department of Education, Food and Nutrition, Parent Teacher Associations, food banks, farming organizations) who can offer support for farm‐to‐school PSE projects?	0.20	“Everything moved a little bit easier” after the school administration and PTA were “kind of seeing the value in that [farm‐to‐school projects].” (CHC Urban Focus Group)
Organizational and practitioner capacity (standardized theme weight = 0.21)†		
**3.1.** To what extent do you have time available each month to seek out or connect with community stakeholders such as agricultural coordinators, school cafeteria managers, or school wellness committees to increase support for implementation of farm‐to‐school PSE projects?	0.30	“…just the time commitment, to maintain it and keep it up, I would say would be a big potential barrier or obstacle.” (CHC Urban Interview)
**3.2.** To what extent does your organization have capacity and tools to evaluate implementation of farm‐to‐school PSE projects in your service area?	0.27	Process evaluation may include efforts to do “surveys with the students to see what they want to see in the salad bar or what to, what should be grown [in the school garden]” while outcome evaluations may include “post surveys to see how they [students] like, how the process went through and whether or not they like it or not.” (CHC Rural Interview)
**3.3.** To what extent does your current organizational or program budget have sufficient funds to support implementation of farm‐to‐school PSE projects in your service area?	0.25	“they [the public schools] will bring us in when the school gets a grant but then once that money's gone and we're gone the garden stops…”(CHC Urban Focus Group)
**3.4.** To what extent does your organization have the capacity to identify, write, and/or submit grants to support implementation of farm‐to‐school PSE projects?	0.18	“I didn't have anything directly to do with that, but it [a non‐profit organization] did have a grant.” (SNAP‐Ed Urban Interview)
Community resources and motivations (standardized theme weight = 0.13)†		
**4.1.** To what extent are parents and students in your service area aware of farm‐to‐school PSE project opportunities such as school gardens, using local foods in meals and snacks (including salad bars), and food and agriculture education at school?	0.28	“You're teaching the next generation and they're bringing it home to their families.” (CHC Urban Focus Group)
**4.2.** To what extent are community leaders in your service area aware of farm‐to‐school PSE project opportunities such as school gardens, using local foods in meals and snacks (including salad bars), and food and agriculture education at school?	0.28	“I think a lot of our community leaders don't know what farm‐to‐school is.” (CHC Urban Focus Group)
**4.3.** To what extent is there momentum or activity among leaders in your service area to address policies or practices (eg, healthy school food environment, standardized testing requirements, and food service regulations) influencing farm‐to‐school PSE projects?	0.24	“That [food service guideline] reflects how the leaderships in schools will react cause if there is no support then there is no sense in doing it [farm‐to‐school]... you just can't overcome that if it's a regulatory requirement.” (CHC Rural Focus Group)
**4.4.** To what extent are farmers in your service area motivated to participate in farm‐to‐school PSE projects?	0.20	“So can they [farmers] even produce enough for [school district name] on a typical school day? Probably not. Can they produce enough for a small project that they were trying to do? Yes. But production is probably the biggest issue. I mean farm‐to‐school is wonderful, but it is a vicious cycle that the farmers are reluctant to produce more if they do not know they can sell it.” (CHC Urban Focus Group)

Responses range from *not at all* (0) to *extremely* (5) with a *do not know* (6) option.

† Standardized final theme weights derived from consensus conference in a range from 0 to 1.

‡ Standardized final indicator weights derived from consensus conference in a range from 0 to 1.

### Theme 1: School Capacity

The school capacity theme was ranked the highest by the expert panel in terms of perceived importance for successful implementation of farm‐to‐school. This theme refers to resources of schools to support implementation of farm‐to‐school projects. Of the 5 indicators related to school capacity, the indicator that was given the highest weight focused on school food service guidelines (Indicator 1.1). Such guidelines were identified as a major barrier to implementing farm‐to‐school. The degree to which farm‐to‐school interventions are integrated into school curricula and activities also seemed to play a critical role in their perceived success and sustainability (Indicator 1.2). The third highest weighted indicator focused on support from teachers, food service staff, custodial staff, and administrators (Indicator 1.3). Participants frequently discussed how important it was to have school staff members who were interested in and/or supportive of farm‐to‐school. The fourth indicator was a concern regarding whether there are supportive programs and resources available to maintain school gardens during summer when schools are typically out (Indicator 1.4). To be sustainable, participants expressed the need for developing supportive programs and resources to maintain efforts during the summer. The last indicator of the school capacity theme related to the availability of paid school staff time to work on farm‐to‐school (Indicator 1.5). Insufficient paid time among school staff including teachers and cafeteria workers was identified as a barrier to farm‐to‐school implementation.

### Theme 2: Networks and Relationships

The second highest weighted theme was networks and relationships, which is defined as the social capital, or the networks of relationships, which practitioners and community members can draw on to implement and support farm‐to‐school. Four indicators were derived to represent the networks and relationships theme. The highest weighted indicator was related to champions for farm‐to‐school (Indicator 2.1). Participants felt that having someone who is passionate about farm‐to‐school can be a facilitator for successful implementation. Two indicators addressed food production, processing, and distribution factors related to farm‐to‐school and these were equally weighted. The first focused on sourcing and aggregation systems (Indicator 2.2). The second indicator focused on distribution systems available to support access to locally produced food items by schools (Indicator 2.3). There was a common belief among participants that local capacity to source and aggregate as well as distribute foods to serve the needs of schools was challenging. The fourth indicator was the degree to which practitioners are connected to or partnered with organizations and/or key personnel who is supportive of farm‐to‐school (Indicator 2.4). It was evident that the enhanced partnerships can facilitate farm‐to‐school implementation among practitioners.

### Theme 3: Organizational and Practitioner Capacity

The third highest weighted theme focused on organizational and practitioner capacity. This theme can be defined as the skills, resources, and capacity of organizations and practitioners to support implementation of farm‐to‐school. The most important indicator of the 4 indicators within this theme focused on the degree to which organizations support the use of practitioner time and resources to build relationships with community stakeholders to increase support for implementation of farm‐to‐school (Indicator 3.1). Specific community stakeholders identified as key resources for these partnerships included agricultural coordinators, school nurses, school cafeteria managers, and school wellness committees. The qualitative findings suggest that the time involved in networking, however, is perceived as a barrier. The second highest weighted indicator was related to the skills needed to evaluate implementation of farm‐to‐school (Indicator 3.2). As described in Table 1, the need for both process and outcome evaluation skills was identified as being important. The third and fourth ranked indicators focused on the financial resources needed to support farm‐to‐school. Indicator 3.3 addressed the availability of organizational funds budgeted to support farm‐to‐school projects. Funding for farm‐to‐school was described as being unstable. Similarly, the fourth indicator was concerned about whether organizations could apply for grants to support implementation of farm‐to‐school (Indicator 3.4). Seeking grants also included strategies of partnering across organizations to support farm‐to‐school.

### Theme 4: Community Resources and Motivations

The fourth theme for implementing farm‐to‐school focused on community resources and motivations. This theme refers to community factors that influence implementation of farm‐to‐school projects. Of the 4 indicators related to community resources and motivations, the first highest weighted indicator focused on awareness and support from parents and students for farm‐to‐school (Indicator 4.1). Participants discussed lack of parental support as a barrier for implementing new programming such as farm‐to‐school. Participants mentioned awareness of farm‐to‐school among parents can be raised by students who participate in these activities. Receiving the same weight was Indicator 4.2 that focused on the degree to which community leaders are aware of farm‐to‐school. Participants frequently expressed that leaders in their communities tended to be unaware of farm‐to‐school which can be a barrier to successful implementation. The indicator with the second highest weight was about momentum or activity among leaders to address policies or practices influencing farm‐to‐school (Indicator 4.3). The findings suggest that the role of community leaders is crucial, particularly to overcome existing barriers such as food service regulations. The last indicator was about farmers' motivations to participate in farm‐to‐school (Indicator 4.4). As described earlier, farmers can be key to the success of farm‐to‐school because they can be a major source of locally grown foods in schools.

## DISCUSSION

In response to dramatic rates of childhood obesity over the past few decades, nutrition‐related interventions such as farm‐to‐school are recommended as community‐level strategies to improve dietary behaviors among school‐aged children. Implementation of farm‐to‐school is influenced by multiple challenges in real‐world settings.^20^, ^21^, ^22^, ^23^, ^24^ It is crucial that front‐line practitioners working in diverse contexts are equipped with solid understanding of the realities of community readiness and capacity needed to support farm‐to‐school implementation.^27^

The findings of this study suggest that there are multiple stakeholders involved in the process of farm‐to‐school implementation. They included teachers and school administrators, nurse and cafeteria manager, school board members and superintendent, parents and students, community leaders, food distributors, and farmers. More importantly, the results suggest that increased awareness of, motivation for, and/or support of farm‐to‐school by these stakeholders can facilitate farm‐to‐school implementation. Furthermore, this study found collaboration and partnership between practitioners, and key community stakeholders was critically needed for farm‐to‐school to be successful.

The availability of support systems across various levels is also viewed as essential. At the individual level, school staff including teachers and cafeteria managers tended to have limited time available to support farm‐to‐school, which could be a major barrier to successful implementation. For practitioners, the availability of time and resources to connect with key community stakeholders to enact necessary partnerships was perceived as an important factor to increase support for farm‐to‐school implementation. At the school level, supportive programs and activities were necessary to maintain farm‐to‐school efforts such as school gardens, particularly when schools were out of session in the summer. Additionally, integration of the interventions into school curriculum and activities was identified as a key factor of successful implementation of farm‐to‐school, as supported by Graham and Zidenberg‐Cherr^24^ in their study with 592 teachers in California.

At the organizational level, the availability of tools and skills to evaluate farm‐to‐school was identified as being important. This result suggests that the evaluation tools and skills can be beneficial both at the implementation and post‐implementation stages. It is not surprising that financial resources emerged as a clear threat to the implementation and sustainability of farm‐to‐school. Both budget allocation and the ability to apply for grants to support farm‐to‐school were identified as major barriers given unstable financial circumstances among public and private entities

At the policy level, school food service guidelines were described as a barrier to farm‐to‐school implementation. This finding is consistent with extant literature. For example, Colasanti et al^22^ found in their study with food service directors of National School Lunch programs in Michigan that federal and state procurement regulations as well as internal purchasing policies were top barriers to farm‐to‐school. In addition, results of the present study identified a significant role of sourcing and aggregation systems as well as distribution systems to support access to locally produced foods at schools. These findings were corroborated by previous research,^20^, ^22^, ^24^ which has found the availability of regionally based food distributors who partnered with local farmers had the potential to bring more locally grown fresh foods to school cafeterias.

The study findings contribute to the existing literature by identifying facilitators of and barriers to implementation of farm‐to‐school from the perspectives of experienced stakeholders and extends previous research by operationalizing them into measurable indicators for frontline practitioners who are interested in farm‐to‐school implementation. Given the complexity of farm‐to‐school implementation that involves diverse stakeholders and their interactions within multiple systems, thoughtful planning prior to implementing farm‐to‐school interventions is recommended. Unlike most farm‐to‐school toolkits currently available that focus on generalized models of implementation, the PSE READI tool provides an opportunity for tailoring implementation plans to the realities of each local context.

### Limitations

There are several potential limitations in this study. First, the results are specific to the state of Ohio, although findings are largely convergent with results from previous studies.^20^, ^21^, ^22^, ^23^, ^24^, ^35^ Second, the stakeholders interviewed and participated in the consensus conference may not be representative of all perspectives with respect to farm‐to‐school implementation. The use of purposive sampling strategies to engage diverse stakeholders may have reduced these risks. Third, it is possible that omitted facilitators and barriers due to the threshold applied during qualitative analysis and consensus development are also critical to farm‐to‐school implementation.

### Conclusions

Efforts to curtail childhood obesity trends must address multiple environments including the school setting. Farm‐to‐school represents one approach to promote exposure to healthier options with the goal of influencing consumption patterns among children. The assessment tool developed through this research may provide guidance for rural and urban communities seeking to implement farm‐to‐school. The PSE READI is designed to support community teams in their assessment of the unique assets and needs of their setting and to guide tailored implementation of farm‐to‐school activities.

## IMPLICATIONS FOR SCHOOL HEALTH

Increased farm‐to‐school activities, including school gardens have numerous implications for school health. Students at schools with successful farm‐to‐school efforts choose healthier school meals options, exhibit a greater willingness to try new foods, consume more fruits and vegetables at school and at home, consume fewer unhealthy foods and sodas, and more frequently ask their families to make healthier food purchases.^6^, ^15^, ^16^, ^17^, ^18^ As the quality of school meals improves, more students choose to consume school meals, which may enhance families' food security. In addition to dietary improvements, farm‐to‐school activities support enhancements in food service operations, including increased fruit and vegetable offerings, seasonal recipes and waste management policy changes.^36^ Research also suggests that farm‐to‐school activities are associated with expanded awareness of gardening, agriculture, and seasonality, increased opportunities for social and emotional growth, and enhanced overall academic achievement.^37^ Benefits go beyond the students to influencing positive changes in teachers' diets and attitudes about farm‐to‐school related curriculum integration.^24^ Farm‐to‐school activities support improvements among food service staff in their motivation, morale, knowledge and interest in local food preparation and seasonal recipes, and interactions that strengthen classroom and cafeteria connections.^36^

### Human Subjects Approval Statement

The treatment of human participants for this study was reviewed and approved by the University Institutional Board. Institutional Review Board procedures were followed and informed consent was obtained from practitioners and community residents who participated in qualitative data collection.
